# Foods, and How to Use Them

**Published:** 1882-01

**Authors:** 


MONTHLY.

E. H. GIBBS, A. M., M. D., EDITOR.

FOUNDED IN 1854.

### FOODS, AND HOW TO USE THEM.

WHOLESOME, nutritious foods, those that supply the system most readily with what is required to give it vigor and endurance, are those foods that are cheapest and most generally distributed over the earth. These are the cereals and other direct products of vegetation. There is as much nutriment in one pound of beans, wheat or rice as there is in three pounds of beef, mutton or poultry. There is not a sturdier race of people in the world than the Chilians, nor a race capable of greater physical endurance. This fact has been again demonstrated in their war with Peru and Bolivia; a war waged against vastly superior numbers and possessing far greater wealth; yet the Chilians conquered them in their own homes, and still hold and govern these countries.

The food of that class from which soldiers are made consists almost exclusively of beans and wheat. These are abundant and cheap in Chili, while animal food is comparatively dear and beyond the reach of the common people, who are miserably poor. In the cities and villages of Chili, instead of the peanut stands that obstruct the streets of American towns, you see tubs filled with boiled wheat in water, which is served at one cent for a pint bowlful, and a spoon is furnished to eat it with. Bean soup or beans boiled or baked cost no more, and one cent is the regular price for two large bread rolls. Three cents there buys an abundant meal of the most wholesome and nutritious food. This seems a very low price ; and many persons will be surprised to learn that these are about the average prices for such foods in this country. Nearly half the people in the world live almost exclusively on rice. I do not profess to be a vegetarian. I do not believe that it would be entirely safe for a person accustomed all his life to animal food to change his diet at once. Such changes have frequently been followed by serious organic disease, usually of the kidneys.

The Chilians, like many other peoples and tribes, have for ages been accustomed to an almost exclusively vegetable diet, and they are robust and strong. Were the present generation to change to a mixed diet, largely composed of animal food, there can be but little doubt they would suffer by the change, just as the people of this country would suffer by changing to an exclusively vegetable diet.

There can be no doubt, however, that a medium course would be most desirable, both in respect to health and economy. The amount of meat consumed, in this land of abundance, is out of all proportion to the consumption of other foods. If people were to inform themselves better in regard to the properties of various foods, and live up to the knowledge acquired, there would be fewer cases of dyspepsia and kindred diseases. A larger consumption of acid fruits should be encouraged. Among animals there are those that are exclusively carnivorous, as the dog and cat; and those that are exclusively herbivorous as the horse. Nature has provided the former with "canine" teeth with which to tear off and divide flesh so that it may be swallowed; but they have no teeth with which to grind their food; flesh then is their natural food. Horses have "incisors" to cut off the grass, and "grinders" with which to masticate grasses and grain.

Man is provided with the three kinds of teeth, incisors "canine teeth" and "grinding teeth." Nature then plainly indicates that we should live on a variety of foods. Some years ago I boarded a year at a hotel in South America, where the table always abounded in the greatest variety of foods I had ever seen on any table; next to me sat a gentleman of great intelligence and courtly manners, who had traveled in many countries, and who spoke fluently several languages; I noticed that he tested every new dish that came on the table, though he ate only moderately. It is the custom in that country to put cigars on the table, and after dinner the gentlemen remain at table and smoke. After an acquaintance of some months, during which time I never saw him use tobacco, he one day took a cigar and lit it. I said that I was not aware that he smoked. "Oh, yes," said he, "I do everything, but in moderation." I shall never forget this reply or his peculiar tone of voice and manner when he said it. The most important lesson of life, in respect to our physical well being, is expressed in that short sentence. If we could only learn to be moderate in all things we should display the greatest wisdom.

The best bill of fare is one that contains the greatest variety of wholesome foods, properly cooked and served, followed by fresh, ripe fruits, to be eaten leisurely and in "moderation." Many families have a "bill of fare" for each day of the week, and the plan is an excellent one when properly carried out, and contributes much to the health and comfort of the family. It has the advantage of system, instead of the usual "hap-hazard" way of providing for the table. Acid fruits, as oranges or cranberries, should be eaten at dinner. They promote digestion and purify the blood.

There are persons who carry the system of self denial to extremes, and who pretend to think it a great virtue to appease the cravings of hanger with a crust of dry bread or some other inadequate palliative. It is safer to attribute it to ignorance. Similar treatment of a dumb animal would merit and probably receive prompt punishment. Downright gluttony is the more reasonable of the two extremes.

